# The mediation effect of social support between stigma and social alienation in patients with stroke

**DOI:** 10.3389/fpubh.2023.1290177

**Published:** 2023-11-29

**Authors:** Yu Wu, Zeping Yan, Lovel Fornah, Jun Zhao, Shicai Wu

**Affiliations:** ^1^School of Nursing and Rehabilitation, Shandong University, Jinan, Shandong Province, China; ^2^University of Health and Rehabilitation Sciences, Qingdao, Shandong Province, China; ^3^Beijing Boai Hospital, China Rehabilitation Research Center, Capital Medical University, Beijing, China; ^4^Department of Epidemiology, School of Public Health, Shandong University, Jinan, Shandong Province, China; ^5^School of Rehabilitation Medicine, Capital Medical University, Beijing, China; ^6^Department of Neurology, Beijing Boai Hospital, China Rehabilitation Research Center, Capital Medical University, Beijing, China

**Keywords:** stroke, stigma, social support, social alienation, mediation effect

## Abstract

**Background:**

Social alienation is prevalent and causes adverse outcomes in stroke. Previous studies have linked stigma with social alienation. However, little is known about the mechanisms behind this relationship. This study explored the mediation effects of social support between stigma and social alienation.

**Methods:**

A cross-sectional design was used to study 248 patients with stroke admitted to a tertiary rehabilitation hospital in Beijing, China, from December 2022 to July 2023. Patients were assessed using a general information questionnaire, the Stroke Stigma Scale, the Social Support Rating Scale, and the Generalized Social Alienation Scale. The PROCESS macro in SPSS was used to examine the mediation model.

**Results:**

The results showed that stigma has a negative effect on social support (*β* = −0.503, *p*<0.001); stigma has a positive effect on social alienation (*β* = 0.768, *p*<0.001). Social support mediated the relationship between stigma and social alienation, with a mediation effect of 0.131 (95%CI: 0.060, 0.214), and indirect effects accounted for 17.06% of the total effect.

**Conclusion:**

Social support mediated the relationship between stigma and social alienation. These findings suggest that intervention targeting the enhancement of social support may prevent or reduce social alienation among patients with stroke.

## Introduction

Stroke is an acute cerebrovascular disease, which is a group of diseases caused by brain tissue damage due to sudden rupture or obstruction of brain vessels, usually divided into two categories: ischemic stroke (that is, cerebral infarction) and hemorrhagic stroke (that is, cerebral hemorrhage, subarachnoid hemorrhage, etc.) (1). In recent years, the incidence of patients with stroke has shown an increasing trend, which has seriously impacted the physical and mental health of patients (2). The crude incidence rate of stroke in the Chinese population has increased. According to a study conducted, from 1990 to 2019, the crude incidence rate for males increased from 142.26 per 100,000 to 269.17 per 100,000, while for females, it rose from 155.61 per 100,000 to 284.46 per 100,000 (3). Patients with stroke are often accompanied by different degrees of functional impairment (such as motor, perceptual, cognitive, etc.) (4). These problems will result in a sense of alienation in patients with stroke. Social alienation has a negative impact on the everyday work and life of patients with stroke, which is also not conducive to the healing of disease and social integration (5).

Social alienation refers to a psychological and behavioral manifestation of automatic alienation and isolation caused by the potential influence of various reasons on social behavior (6). With stroke, patients often have different degrees of motor impairment, making it difficult to walk and reintegrate into society and negatively affecting their physical and mental health (7). There are few studies on the social alienation of patients with stroke, most of which focus on the negative emotions and motor impairment caused by stroke (8, 9). Therefore, it is essential to investigate the social alienation of patients with stroke and explore its potential mechanism to help patients better adapt to society.

Stigma refers to the shame of patients doubting their own values and creating stereotypes in social groups due to the original disease. Patients with a high degree of stigma can be manifested as having low self-esteem, trying to conceal the disease, consciously avoiding society and negatively coping with rehabilitation training (10). Previous research has examined the phenomenon of stigma in patients with mental illness (11), HIV infection (12), epilepsy (13), tuberculosis (14), and stroke, which is similarly subject to societal stigmatization. Consequently, a growing amount of research has been dedicated to investigating the phenomenon of stigma associated with stroke. In light of the presence of limb dysfunction, cognitive impairment, and other sequelae, patients who have experienced a stroke commonly experience feelings of shame. This emotional response might hinder their participation in social interactions, therefore leading to interpersonal stress.

According to a study conducted by Li Yongping et al. (15), stigma may increase patients’ sense of social alienation. Additionally, social support may alleviate the social alienation of patients, which provides psychological comfort and compensation to patients (16). In other words, social support is an important emotion-focused coping resource to alleviate social alienation among patients with stroke (17). Patients with stroke identify the various changes in appearance and function that occur during the process of stroke treatment and rehabilitation to narrow the gap between themselves and the reality by using their own resources, and actively adapt to society. However, there are currently few investigations on the mechanism of stigma and social alienation in stroke patients. Furthermore, no study has been undertaken to our knowledge to investigate the mediation effects of social support between stigma and social alienation.

### Theory framework

**Figure 1:**
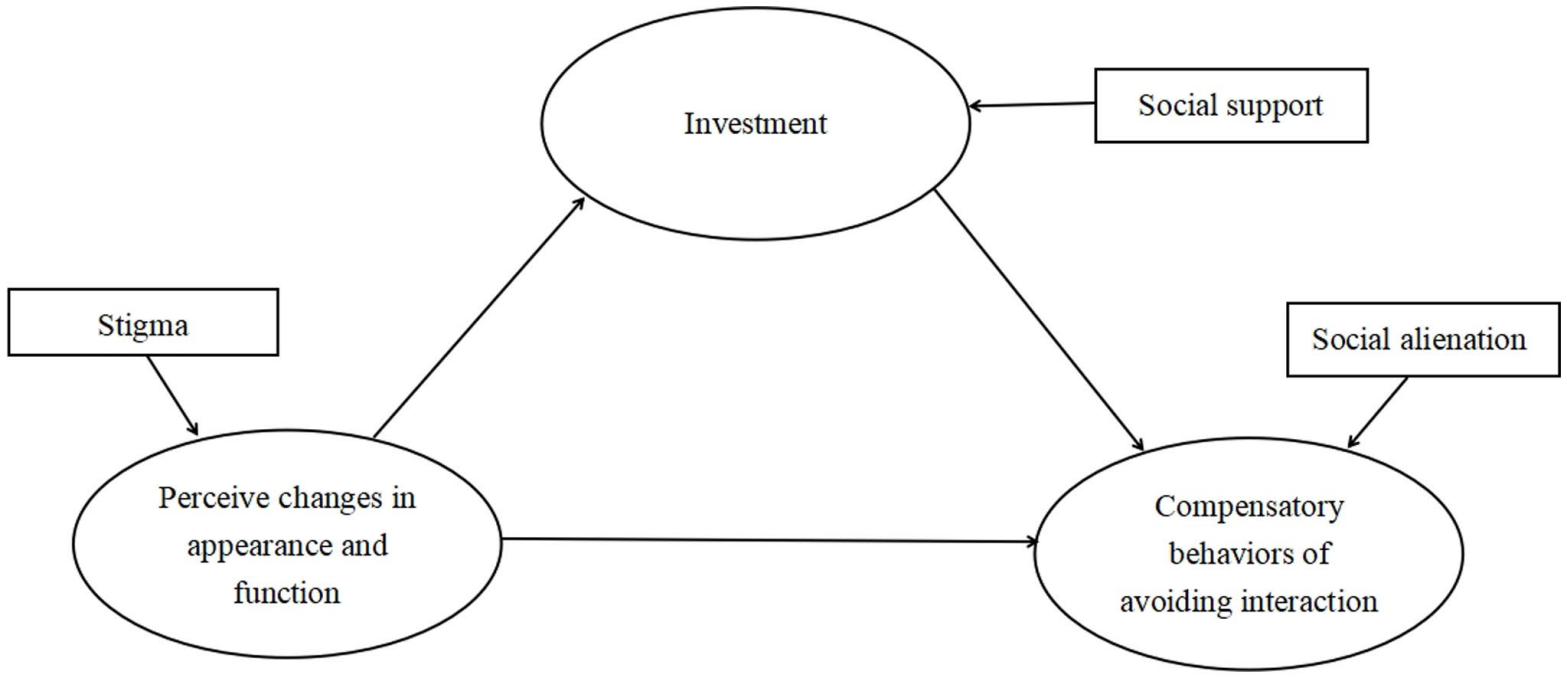
The White heuristic cognitive behavior model is adopted in this study.

The White heuristic cognitive behavior model (18) based on emotional cognition theory points out that when patients perceive changes in appearance and function, they will have the cognition that appearance is everything, and then increase their investment in changing their own characteristics to reshape their new selves. However, when patients find that there is still a gap with reality, they develop negative emotions and compensatory behaviors of avoiding interaction. Therefore, in this study, we uesd the White heuristic cognitive behavior model to explore the mediation effects of social support between stigma and social alienation among patients with stroke by taking stigma as perception of appearance changes, social support as investment, and social alienation as avoidant compensation behavior (Figure 1).

In summary, this study focused on social alienation among patients with stroke, with a particular emphasis on the mechanisms by which stigma affects social alienation. The study hypothesized that social support could mediate the relationship between stigma and social alienation.

## Methods

### Study design and sample

This cross-sectional study aimed to explore the mediation effects of social support between stigma and social alienation. Participants were recruited using convenience sampling at a tertiary rehabilitation hospital in Beijing, China, from December 2022 to July 2023. The study complied with the principles outlined in the Declaration of Helsinki and was approved by the medical ethics committee of the hospital where the participants were located (2022-133-01).

Inclusion criteria included: (1) The diagnosis was made by head Computed Tomography (CT) or Magnetic Resonance Imaging (MRI) and met the diagnostic criteria for stroke formulated by the Fourth National Conference on Cerebrovascular Diseases in 1995 (19); (2) Onset of 1 to 6 months; (3) Cooperated to complete the investigation.

Exclusion criteria included (1) With Cognitive impairment; (2) With speech dysfunction.

Based on the cross-sectional study’s sample size calculation technique, the required sample size was determined to be ten times the maximum number of questionnaire items (20). In this study, the maximum number of items on the Stroke Stigma Scale was 16. Considering the invalid response rate of 20%, the required sample size was 192. In the end, a total of 248 patients were investigated.

## Measures

### General information questionnaire

The researchers compiled the questionnaire’s general information based on literature reviews. The general information included gender, Body Mass Index (BMI), age, level of education, marital status, primary caregiver status, frequency of strokes, type of chronic disease, and average monthly family income.

### Stroke stigma scale

Zhu Minfang developed the Stroke Stigma Scale to evaluate the stigma of patients with stroke (21). The Stroke Stigma Scale comprises four dimensions, namely self-perception (items 1–5), somatic impairment (items 6–9), discrimination experience (items 10–13), and social interaction (items 14–16), totaling 16 items. The Likert 5-level scoring method is utilized by the scale, encompassing a range from “never” to “always” with successive scoring of 1 to 5 points. Notably, items 1, 3, 4, 11, and 14 are reverse-scored. The total score on this scale can vary between 16 and 80 points. A positive correlation exists between a higher score and an elevated level of stigma. The coefficient of Cronbach’s α was determined to be 0.916.

### Social support rating scale

Xiao ShuiYuan developed the Social Support Rating Scale to evaluate the social support level of patients (22). The Social Support Rating Scale comprises three distinct dimensions: objective support (items 1, 3, 4, and 5), subjective support (items 2, 6, and 7), and utilization of support (items 8 to 10). In total, the scale consists of ten items. The scoring methodology for the items in question is as follows: Items 1 to 4 and 8 to 10 are each assigned a score based on a 4-level scale, ranging from 1 to 4 points. Item 5, on the other hand, is subdivided into four distinct dimensions, namely A, B, C, D, and E, with each dimension being evaluated and awarded a score ranging from 1 to 4 points. As for items 6 and 7, a score of 0 point is assigned in cases where no source is present, while a score of 1 point is allocated for each remaining source. The total score spans a range of 12 to 66 points. A positive correlation exists between a higher score and an elevated level of social support. The coefficient of Cronbach’s α was determined to be 0.820 (15).

### Generalized social of alienation scale

The Generalized Social Alienation Scale was developed by Jessor R & Jessor S and translated by Wu Shuang et al. (23) to evaluate individuals’ feelings of isolation and uncertainty regarding participating in various activities. The scale comprises four distinct dimensions, namely social isolation (items 1–5), powerlessness (items 6–9), self-alienation (items 10–12), and meaninglessness (items 13–15), resulting in a comprehensive set of 15 items. The scale utilized in this study is assessed using a 4-point Likert scale, where respondents are asked to indicate their level of agreement or disagreement. The scale ranges from 1 to 4, with a score of 1 corresponding to “strongly disagree,” a score of 2 indicating “disagree,” a score of 3 representing “agree,” and a score of 4 denoting “strongly agree.” The scale’s total score spans a range of 15 to 60 points, wherein a higher score corresponds to a higher sense of social alienation. The Cronbach’s α coefficient for this scale was determined to be 0.81, indicating a high level of internal consistency. Additionally, the split-half reliability coefficient was found to be 0.80. Furthermore, the retest reliability coefficient was calculated to be 0.76 (24, 25). Xiao Ming first compiled it and later translated into Chinese by Wu Shuang et al. (23) evaluated the social alienation of the older adults and showed that Cronbach’s α coefficient was 0.77, which had good reliability.

### Statistical analysis

Statistical data analysis was conducted using SPSS 21.0 and PROCESS 3.3 software. The measurement data for a normal distribution was described using the mean and standard deviation (SD), whereas the measurement data associated with a non-normal distribution were described using the median and quartiles. The quantitative data were analyzed using frequencies and percentages. Pearson correlation analysis was employed to examine the correlation between stigma, social alienation, and social support. The study employed multiple linear regression analysis to investigate the influencing factors of social alienation in stroke patients. The mediation effect of social support between stigma and social alienation was analyzed using Model 4 in the PROCESS 3.3 macro. A significance level of 0.05 was employed consistently throughout the study. The graphs were created using Python and Excel.

## Results

### Patient characteristics and comparison by social alienation

Out of the total participants of 248 individuals diagnosed with stroke, 45 were excluded from the analysis due to incomplete data. Consequently, the present study utilized data from a sample of 203 stroke patients, with a significant questionnaire recovery rate of 81.9%. Table 1 displays the demographic characteristics and social alienation comparisons. In the sample population, 4 individuals (2.0%) were classified as thin, while the majority, consisting of 110 individuals (54.2%), fell within the normal weight range. Additionally, 73 individuals (36%) were categorized as overweight, leaving the remaining participants classified as obese, accounting for 7.9% of the total population. The study revealed that divorced individuals who experienced a stroke exhibited the highest levels of social alienation, with an average score of 40.00 ± 2.83. There were significant differences in the scores of social alienations among patients with different stroke frequencies (*t* = −2.106, *p* < 0.05) and average monthly family income (*F* = 2.905, *p* < 0.05). However, there were no differences in participants’ gender, BMI, age, level of education, marital status, primary caregiver, and types of chronic diseases (*p* > 0.05).

**Table 1:** Single factor analysis of social alienation in patients with stroke with different demographic data (*N* = 203).

Item	Category	*n*/%	Mean ± SD	Statistic	*p*
Gender	Male	147 (72.4)	34.31 ± 5.47	−0.407^(1)^	0.685
	Female	56 (27.6)	34.63 ± 4.80		
BMI	<18.5	4 (2.0)	35.50 ± 6.56	2.486^(2)^	0.062
	18.5 ~ 23.9	110 (54.2)	35.26 ± 4.85		
	24 ~ 27.9	73 (36.0)	33.36 ± 5.62		
	≥28	16 (7.9)	32.88 ± 5.60		
Age	<60	84 (41.4)	34.36 ± 5.03	0.324^(2)^	0.808
	60 ~ 69	82 (40.4)	34.73 ± 5.33		
	70 ~ 79	32 (15.8)	33.81 ± 5.74		
	≥80	5 (2.5)	33.20 ± 6.91		
Level of education	Primary school	7 (3.4)	37.29 ± 5.22	0.711	0.585
	Junior high school	28 (13.8)	33.96 ± 6.34		
	Senior high school and technical secondary school	85 (41.9)	34.60 ± 5.23		
	Undergraduate and Junior College	70 (34.5)	34.19 ± 4.61		
	Graduate	13 (6.4)	33.54 ± 6.72		
Marital status	Single	10 (4.9)	35.10 ± 4.61	1.262^(2)^	0.288
	Married	189 (93.1)	34.25 ± 5.32		
	Widowed	2 (1.0)	38.50 ± 3.54		
	Divorced	2 (1.0)	40.00 ± 2.83		
Primary caregiver	Spouse	64 (31.5)	34.17 ± 5.27	0.175^(2)^	0.913
	Offspring	12 (5.9)	34.00 ± 5.06		
	Nursing assistant	104 (51.2)	34.65 ± 5.45		
	Others	23 (11.3)	34.04 ± 4.94		
Stroke frequency	One	178 (87.7)	34.16 ± 5.40	**−2.106** ^**(1)**^	**0.042**
	Two or more	25 (12.3)	36.08 ± 4.09		
Type of chronic diseases	None	48 (23.6)	33.58 ± 5.23	1.372^(2)^	0.256
	One	132 (65.0)	34.45 ± 5.30		
	Two or more	23 (11.3)	35.78 ± 5.22		
Average monthly family income	1,001 ~ 3,000	3 (1.5)	36.67 ± 0.58	**2.905** ^**(2)**^	**0.036**
	3,001 ~ 5,000	17 (8.4)	37.12 ± 5.09		
	5,001 ~ 7,000	55 (27.1)	35.11 ± 5.13		
	>7,000	128 (63.1)	34.39 ± 5.28		

(1) t; (2) F. Abbreviations: Standard Deviation. Bold values indicate statistical significance.

### Scores of stigma, social support, and social alienation in patients with stroke

**Figure 2:**
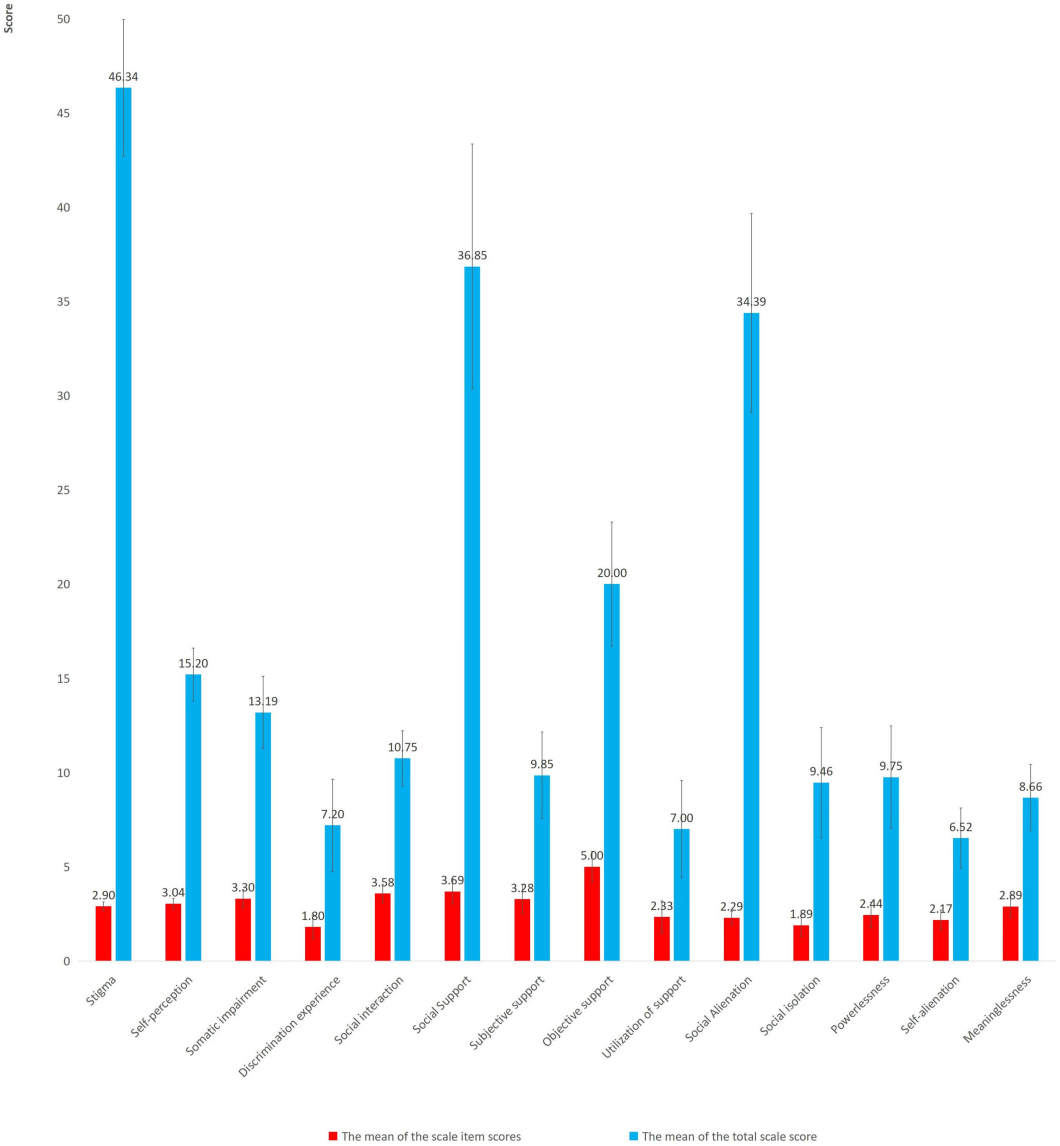
The scores of stigma, social support and social alienation in patients with stroke.

Figure 2 presents the scores of stigma, social support, and social alienation in patients with stroke. The average stigma score was 46.34 (SD = 3.63), and the score of the self-perception dimension was the highest (15.20 ± 1.41). The social support score in patients with stroke was 36.85 (SD = 6.50), and the score of subjective support dimension was the highest (20.00 ± 3.29). The average score of social alienation was 34.39 (SD = 5.28), and the score of powerlessness was the highest (9.75 ± 2.71).

### Correlation analysis of stigma, social support, and social alienation in patients with stroke

**Figure 3:**
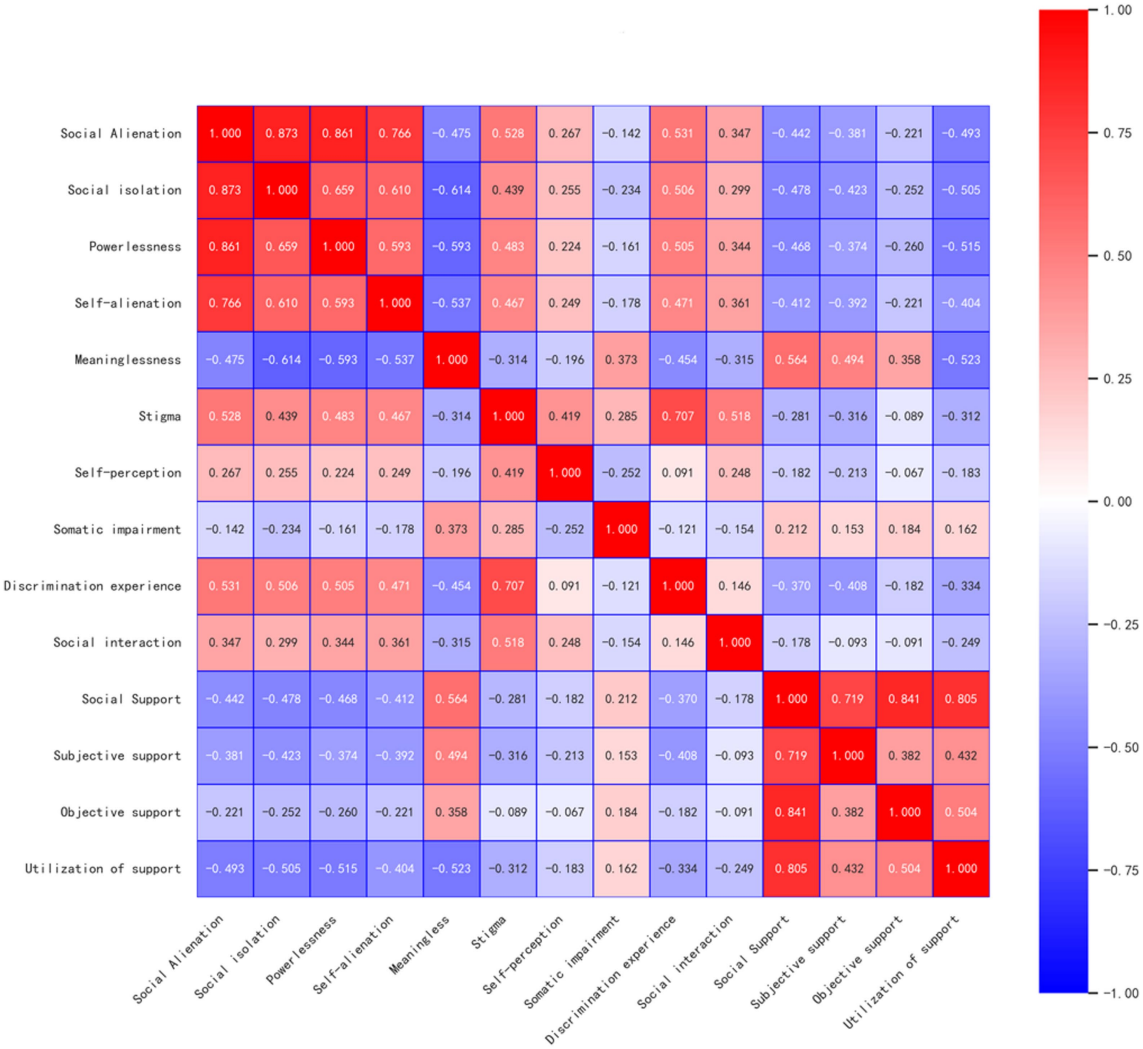
The correlation analysis of stigma, social support and social alienation.

Figure 3 presents the result of the correlation analysis. The total score for social alienation was positively correlated with the score for stigma (*r* = 0.528, *p* < 0.001) and negatively correlated with the score for social support (*r* = −0.442, *p* < 0.001).

### Multiple linear stepwise regression analysis of social alienation in patients with stroke

Multiple linear stepwise regression analysis was performed with social alienation as the dependent variable, stigma and social support as the independent variable, and stroke frequency and average monthly family income as the control variables. This study compared the demographic characteristics with social alienation and found that stroke frequency (*t* = −2.106, *p* < 0.05) and average monthly family income (*F* = 2.905, *p* < 0.05) had an impact on social alienation. The results of the study by Yang Xumeng et al. (16) showed that stroke frequency and average monthly family income had an important impact on social alienation. To accurately assess the association of stigma and social support with social alienation, stroke frequency and average monthly family income were used as control variables. The results showed that both stigma and social support were included in the regression equation, explaining 36.6% of the total variation in social alienation among patients with stroke, as shown in Table 2.

**Table 2:** Multiple linear stepwise regression analysis of influencing factors of social alienation in patients with stroke.

Item	β	SE	β’	*t*	*p*
constant	14.408	4.743	—	3.038	0.003
Stigma	0.638	0.085	0.438	7.508	<0.001
Social support	−0.259	0.047	−0.319	−5.471	<0.001

*R*^2^ = 0.373, Adjusted *R*^2^ = 0.366, F = 59.394, *p* < 0.001. SE = Standard error.

### The analysis of mediation effect of social support

**Figure 4:**
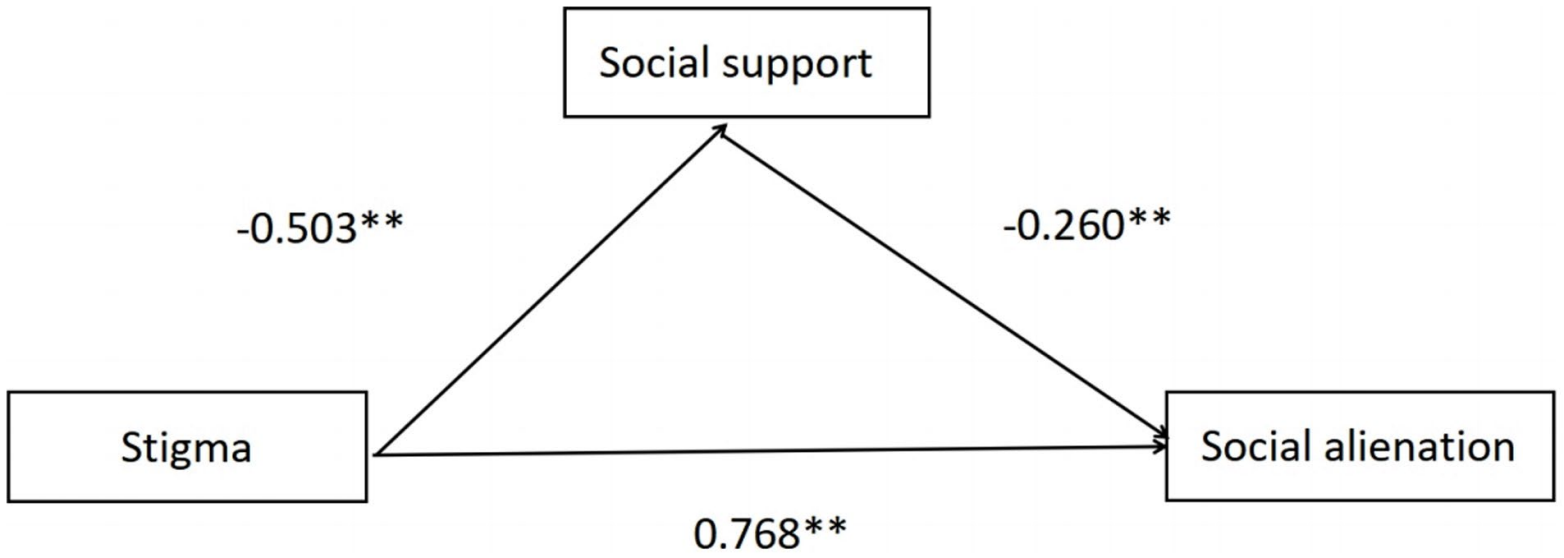
The relationship between stigma and social alienation: mediation effects of social support.

The results of the mediation effect analysis showed stigma could significantly positively predict social alienation (*β* = 0.768, *p*<0.001); stigma could significantly negatively predict social support (*β* = −0.503, *p*<0.001) (Figure 4). The Bootstrap sampling test method was used to conduct a study on the mediation effect of social support between stigma and social alienation, and the sampling times were 5,000 times. The results showed that the mediation effect of social support was tested with a 95% confidence interval (CI) excluding 0 (Bootstrap 95% CI: 0.060, 0.214), indicating that stigma could affect social alienation through the mediation effect of social support and the mediation effect of social support accounts for 17.06% of the total effect, as shown in Table 3.

**Table 3:** The mediation effect of social support between stigma and social alienation.

Paths	Effect	SE	95%CI		%
			LLCI	ULCI	
Total effect (C)	0.768	0.087	0.596	0.940	
Direct effects (c’)	0.638	0.085	0.470	0.805	83.07
Total indirect effect (a × b)	0.131	0.039	0.060	0.214	17.06

SE, Standard Error; CI, Confidence interval; LLCI, Lower Limit Confidence interval; ULCI, Upper Limit Confidence interval.

## Discussion

The present study utilized a cross-sectional design to investigate the degree of social alienation and the factors that influence social alienation among stroke patients. The study’s findings revealed that patients with stroke exhibited a social alienation score of 34.39 (SD = 5.28), indicating an intermediate level of social alienation. The findings align with the research outcomes reported by Zhao Cuicui et al. (6). One of the reasons may be that stroke patients are accompanied by family members or nursing assistants who can give them specific psychological support. The second possibility is that stroke patients have different degrees of motor impairment, resulting in low self-esteem and a certain sense of social alienation.

Therefore, the result of multiple linear stepwise regression showed that stigma and social support were associated with the level of social alienation in stroke patients. The results showed that the higher the level of stigma, the higher the score of social alienation. This result is similar to those of Zhang Lin et al. (26). Moreover, stigma is a kind of psychological stress experience caused by disease, which increases the psychological pressure on patients and leads to their own social maladjustment. First of all, the nursing team should pay attention to psychological nursing and know the needs and feelings of patients to help patients enhance their self-confidence. In addition, the mental toughness of stroke patients can be enhanced by adopting regular positive coping strategies (27). For example, the medical staff of community service centers should regularly carry out popular science propaganda to help stroke patients correctly understand the disease (28), thereby reducing stigma.

In addition, the results showed that stroke patients with higher social support had lower feelings of social alienation (29). This may be related to patients with high levels of social support having higher levels of social integration and participation. A study by Hinzey et al. (30) showed that patients could reduce their perception of social alienation by acquiring additional social support, including resources, information, and emotional assistance. It is recommended to use peer support intervention to help patients gain a sense of belonging and enhance the level of social support to alleviate social alienation.

The negative association between stigma and social support observed in this study aligns with the findings reported by Xu Shan et al. (31). This study found that social support plays a partial mediating role between stigma and social alienation. In order to return to society as soon as possible, patients with low levels of stigma often take the initiative to seek social support to help them accept the impact of the disease, promote their participation in social activities after stroke, and reduce social alienation (32). However, due to high levels of stigma, patients with stroke have social avoidance and are reluctant to communicate with others, resulting in reduced social support and increased social alienation. A better family atmosphere is conducive to helping patients actively participate in social activities and reducing patients’ sense of social alienation (33). Therefore, increasing social support may be an intervention strategy to prevent or reduce social alienation among stroke patients, especially those with higher stigma. Our study clearly indicated the mediating role of social support in understanding why patients with higher stigma are less likely to social alienation.

It is essential to acknowledge that certain limitations exist in this study’s scope. First, using a study sample derived solely from a single medical hospital in the capital of China may yield findings that lack generalizability to the broader population of stroke patients. Conducting multi-center and multi-regional studies are imperative in order to ensure the generalizability of findings. Second, owing to the study’s cross-sectional nature, our inferences are limited to statistical associations rather than establishing causal relationships. The verification of causality can be deemed necessary by conducting longitudinal studies. Ultimately, while the present study possessed an adequate sample size, it is recommended that future investigations be conducted with a more expansive sample size in order to ensure reproducibility.

However, our investigation offers significant contributions to the study of social alienation in individuals diagnosed with stroke. First and foremost, it appears that social alienation is greatly affected by the presence of stigma and a sense of social support. Additionally, it has been observed that social support plays a significant mediating role in this process, mitigating the effect of stigma. Finally, these interventions, focusing on reducing stigma, indirectly prevent or reduce social alienation by enhancing social support.

## Conclusion

Managing social alienation is of great importance to patients with stroke. Interventions could be designed to target the enhancement of social support based on their mediation effects on the relationship between stigma and social alienation to manage social alienation among stroke patients with high levels of stigma.

## Data availability statement

The raw data supporting the conclusions of this article will be made available by the authors, without undue reservation.

## Ethics statement

The studies involving humans were approved by Medical Ethics Committee of China Rehabilitation Research Center. The studies were conducted in accordance with the local legislation and institutional requirements. The participants provided their written informed consent to participate in this study.

## Author contributions

YW: Data curation, Investigation, Methodology, Writing – original draft, Writing – review & editing. ZY: Data curation, Investigation, Writing – original draft, Writing – review & editing. LF: Data curation, Investigation, Writing – original draft, Writing – review & editing. JZ: Funding acquisition, Supervision, Writing – review & editing. SW: Funding acquisition, Supervision, Writing – review & editing.
